# Intra- and Interobserver Reliability of the CT-Based Glenoid Arc-Angle Method Using Injured-Side and Contralateral Reference Circles: A Diagnostic Reliability Study

**DOI:** 10.3390/diagnostics16152477

**Published:** 2026-08-06

**Authors:** Susanne Strasser, Johannes Dominikus Pallua, Anton Aschaber, Franz Kralinger, Dietmar Dammerer, Dietmar Krappinger, Rohit Arora, Clemens Hengg

**Affiliations:** 1 Department of Orthopaedics and Traumatology, Medical University Innsbruck, 6020 Innsbruck, Austria; susanne.kathrein@i-med.ac.at (S.S.); anton.aschaber@gmail.com (A.A.); dietmar.krappinger@i-med.ac.at (D.K.); rohit.arora@i-med.ac.at (R.A.); clemens.hengg@tirol-kliniken.at (C.H.); 2 Department for Orthopaedics and Traumatology, Hospital Ottakring, 1160 Wien, Austria; franz.kralinger@gesundheitsverbund.at; 3 Department for Orthopaedics and Traumatology, University Hospital Krems, 3500 Krems, Austria; dietmar.dammerer@krems.lknoe.at

**Keywords:** anterior shoulder instability, glenoid bone loss, computed tomography, contralateral glenoid, reliability, preoperative assessment

## Abstract

**Background/Objectives**: This study aimed to compare the intraobserver and interobserver reliability and absolute agreement of the CT-based glenoid arc-angle method when the reference circle was constructed either directly on the injured glenoid or transferred from the healthy contralateral glenoid. The objective was to assess measurement reproducibility rather than anatomical validity, diagnostic accuracy, or clinical utility. **Methods**: Preoperative CT scans of 31 patients with anterior shoulder instability who underwent surgery were retrospectively analyzed. Three independent observers with different levels of experience performed measurements at two separate time points using two geometrical approaches. In the contralateral-reference method, the diameter of the best-fit circle was determined on the healthy contralateral glenoid and transferred to the injured side. In the injured-side method, the best-fit circle was determined directly on the injured glenoid. The defect angle and glenoid diameter were measured, and the defect angle was converted into an area-based percentage of glenoid bone loss. Intraobserver reliability was assessed across the two measurement sessions, whereas interobserver reliability was assessed across observer-specific means from the two sessions, using intraclass correlation coefficients with 95% confidence intervals. Absolute agreement, systematic bias, and measurement error between sessions were additionally evaluated using Bland–Altman analysis, the standard error of measurement, and the minimum detectable change. **Results**: The contralateral-reference method yielded numerically higher ICC point estimates for both measured parameters. For defect-angle measurements, the interobserver ICC based on observer-specific two-session mean estimates was 0.890 with the contralateral-reference method (95% CI: 0.811–0.942) and 0.603 with the injured-side method (95% CI: 0.399–0.767). For glenoid-diameter measurements, the corresponding interobserver ICCs were 0.900 (95% CI: 0.660–0.961) and 0.812 (95% CI: 0.672–0.900), respectively. Several confidence intervals crossed conventional reliability category boundaries and partially overlapped between methods; therefore, these findings represent numerical differences in point estimates rather than statistically established superiority. **Conclusions**: Within this cohort, the contralateral-reference method yielded numerically higher ICC point estimates, smaller session-related biases, and lower measurement-error estimates than the injured-side method. These descriptive findings do not establish statistically significant superiority, diagnostic accuracy, clinical utility, or treatment thresholds. The results are limited by the relatively small retrospective cohort of 31 surgically treated patients and the absence of anatomical or clinical outcome validation.

## 1. Introduction

Both static and dynamic stabilizers maintain the stability of the glenohumeral joint. Static stabilizers include the glenoid labrum, capsule, articular surfaces, and glenohumeral ligaments, whereas dynamic stabilizers include the rotator cuff muscles and the long head of the biceps tendon. Owing to its wide range of motion and relatively shallow glenoid socket, the glenohumeral joint is particularly prone to subluxation and dislocation. Zacchilli et al. reported that the shoulder is among the most frequently dislocated joints in the human body [1]. Glenoid bone loss is a common consequence of anterior shoulder instability and has important implications for surgical treatment. Bois et al. reported that glenoid bone loss is present in 41% of patients after a first-time dislocation, in 90% of patients with recurrent instability, and in 89% of patients who have undergone failed arthroscopic soft-tissue stabilization [2]. Several surgical treatment options have been described for the management of anterior shoulder instability associated with osseous defects, including Resch’s J-bone graft, the Eden–Hybinette procedure, the Latarjet procedure, and open Bankart repair [3,4]. The extent of glenoid bone loss influences the choice of surgical technique. Previous studies have suggested critical thresholds for glenoid bone loss of approximately 20–25%, depending on the measurement technique used, including the superoinferior glenoid length, the diameter of the inferior glenoid best-fit circle, or the bare spot as the center of the glenoid [5]. Historically, glenoid bone loss of approximately 20–25% has been considered “critical,” as defects within this range are associated with an increased risk of failure after isolated soft-tissue stabilization. However, Shaha et al. demonstrated worsening functional outcomes at approximately 13.5% bone loss, despite values remaining below the traditional critical threshold. This finding contributed to the concept of “subcritical” glenoid bone loss and indicates that accurate quantification may be clinically relevant even for defects below 20–25% [6]. Accurate quantification of glenoid bone loss is therefore essential, as osseous deficiency is one of the most important predictors of failure after soft-tissue stabilization and strongly influences preoperative planning [7,8]. A variety of imaging modalities and measurement techniques have been proposed for assessing glenoid bone loss [9,10,11,12,13,14]. Computed tomography (CT) is currently regarded as one of the most reliable imaging modalities for evaluating osseous glenoid defects [15]. Moreover, previous studies have shown that three-dimensional CT does not provide a clear advantage over carefully reconstructed two-dimensional CT for this purpose [4]. Despite the wide range of available methods, no universally accepted gold standard currently exists for the preoperative quantification of glenoid bone loss in patients with anterior glenohumeral instability [2].

Best-fit-circle methods reconstruct the presumed native glenoid contour either from the residual contour of the injured glenoid or by reference to the unaffected contralateral side. When the anterior glenoid rim is substantially disrupted, positioning and sizing a best-fit circle directly on the injured side may require extrapolation from an incomplete cortical contour. This may increase observer-dependent variation, particularly when the preserved inferior or posterior rim is irregular or only partially visible. In contrast, the unaffected contralateral glenoid provides an intact anatomical contour from which the native circle diameter can be determined before transfer to the injured side.

Contralateral referencing is an established concept. Kuberakani et al. compared an injured-side best-fit-circle method with a contralateral comparison method in 94 patients using humeral-head-subtracted three-dimensional CT reconstructions and reported higher intraobserver and interobserver reliability for the contralateral method [16]. Milano et al. evaluated 200 patients using two-dimensional and three-dimensional CT measurements performed with and without comparison with the contralateral shoulder and reported very good agreement among the four approaches [17]. However, these studies evaluated different geometric outcomes and reconstruction workflows. The glenoid arc-angle method derives an area-based defect estimate from the central angle formed by the anterior defect chord and the center of the reference circle [18,19]. The reproducibility and absolute agreement of this method when the reference circle is constructed either on the injured glenoid or transferred from the unaffected contralateral glenoid have not been sufficiently characterized using repeated measurements by observers with different levels of experience and routine two-dimensional multiplanar reconstructions. The present study therefore does not introduce contralateral referencing as a novel concept. Instead, it provides a method-specific assessment of the reproducibility and absolute agreement of the CT-based glenoid arc-angle method. The primary objective was to compare the intraobserver and interobserver reliability and absolute agreement of defect-angle, glenoid-diameter, and calculated percentage bone-loss measurements obtained using an injured-side reference circle and a contralateral reference circle. The study was designed to assess measurement reproducibility and did not evaluate anatomical validity, diagnostic accuracy, clinical utility, treatment selection, or patient outcomes.

## 2. Materials and Methods

### 2.1. Study Design, Relationship to Previous Work, and Sample Size Considerations

This retrospective study was designed as a Level III exploratory diagnostic reliability study evaluating the reproducibility and absolute agreement of the CT-based glenoid arc-angle method. The study compared two approaches for defining the reference circle: construction of the circle directly on the injured glenoid and transfer of a reference circle derived from the unaffected contralateral glenoid. The objective was to assess measurement reproducibility and absolute agreement rather than anatomical validity, diagnostic accuracy, clinical superiority, therapeutic utility, or associations with patient outcomes. The patient cohort and the original repeated observer measurements were generated as part of an academic thesis completed in 2014. These data have not previously been published as a peer-reviewed original research article. Members of the present author group also published a correspondence in 2014 concerning the geometric description and attribution of the glenoid arc-angle method [19]. That correspondence did not present the complete patient-level measurements or the observer-specific reliability and agreement analyses reported in the present manuscript. The current study provides an expanded analysis including ICC estimates with 95% confidence intervals, calculated percentage glenoid bone-loss values, Bland–Altman analyses, standard errors of measurement, and minimum detectable changes. No formal a priori sample-size calculation based on predefined acceptable and expected ICC values was performed. The original academic project used a pragmatic target of at least 30 patients. More than 500 archived CT examinations were available for screening, from which 31 patients treated between 2007 and 2012 were selected according to the eligibility criteria. The present investigation should therefore be regarded as exploratory rather than as a prospectively powered confirmatory reliability study. All reliability estimates are reported with 95% confidence intervals to indicate their precision and statistical uncertainty [20,21,22].

### 2.2. Cohort and Inclusion Criteria

More than 500 archived CT examinations were available for retrospective screening. From these examinations, 31 patients treated for recurrent anterior shoulder instability between 2007 and 2012 were selected according to the predefined eligibility criteria and included in the measurement analysis. Eligibility required bilateral CT imaging performed before the first surgical stabilization procedure, no previous surgery involving either shoulder, and subsequent treatment with either a J-bone graft procedure or screw fixation of an osseous glenoid fragment. The institutional ethics review board approved the study before inclusion of the CT data.

### 2.3. Exclusion Criteria

Patients were excluded if they had undergone previous surgery involving either shoulder, if bilateral CT imaging before the first stabilization procedure was unavailable, or if they were subsequently treated with a procedure other than a J-bone graft or screw fixation of an osseous glenoid fragment. Exact numbers for the individual exclusion reasons could not be reconstructed from the archived study documentation.

### 2.4. Data Collection Methods

All included patients subsequently underwent either a J-bone graft procedure or screw fixation of an osseous glenoid fragment. For each patient, age at diagnosis, sex, affected-side laterality, and the type of surgical procedure were recorded.

#### 2.4.1. Diagnostic Imaging

Preoperative CT examinations of both shoulders were acquired according to the institutional imaging protocol using a LightSpeed VCT scanner (GE Medical Systems, Milwaukee, WI, USA). Patients were positioned head-first supine, with both shoulders in neutral rotation. Following acquisition of a scout image, both shoulders were included in a single helical acquisition. Available archived protocol documentation indicated that both shoulders were included in a bilateral helical acquisition. The scan range extended cranially from below the orbits to approximately 10 cm caudal to the humeral heads. The axial source images were reconstructed with a nominal slice thickness of 0.62 mm. Tube voltage, tube current, reconstruction increment, in-plane pixel spacing, reconstruction field of view, reconstruction kernel, and image matrix could not be reconstructed reliably for the complete cohort from the available archived documentation and are therefore not reported. Multiplanar two-dimensional reconstructions were generated from the primary axial CT data using IMPAX software (IMPAX EE R20 XVIII SU1; AGFA HealthCare N.V., Mortsel, Belgium). At each measurement session, every observer independently generated the standardized en-face reconstruction from the axial source images. The reconstruction plane was adjusted in orthogonal orientations using the anterior, posterior, superior, and inferior glenoid rim contours as anatomical landmarks. A true en-face view was defined as an image in which the complete glenoid rim was visible without apparent rotation or foreshortening. The humeral head was not digitally removed. Consequently, the reported reliability incorporates variability arising from both the selection of the multiplanar reconstruction plane and the subsequent geometrical measurements. Three independent observers performed all measurements at two separate measurement sessions. The observers had different levels of experience and included one experienced shoulder surgeon, one final-year medical student, and one junior medical student. Before the first formal measurement session, the experienced observer trained the two less experienced observers in the application of the measurement method. The training covered generation of the en-face reconstruction, positioning of the best-fit circle, identification of the defect margins, placement of the anterior defect line, and measurement of the defect angle. Written instructions documenting the measurement procedure were provided at the time of the study; however, these instructions were not retained as a formal study manual. No feedback or consensus review was provided during the formal measurement sessions. A minimum interval of four weeks was maintained between the two sessions. Measurements were performed independently, and the observers were blinded to the measurements of the other observers and to their own previous results.

#### 2.4.2. Anatomical Measurements

Two geometrical measurement methods were evaluated.

Before performing the geometric measurements, the observer confirmed that the complete glenoid rim was displayed in an orthogonal en-face orientation without visible foreshortening. The best-fit circle was positioned over the inferior portion of the glenoid and adjusted to obtain maximum congruence with the preserved cortical rim. On the healthy contralateral side, the circle was fitted to the intact inferior, anterior, and posterior cortical contours. On the injured side, the anterior defect margin was excluded when determining the circle size and position; the circle was fitted primarily to the preserved inferior and posterior cortical contours. For the contralateral-reference method, the diameter of the best-fit circle determined on the unaffected glenoid was transferred to the injured glenoid and was not resized after transfer. The transferred circle was positioned using the preserved inferior and posterior cortical contours. The anterior defect line was drawn through the superior and inferior cortical transition points delimiting the anterior bone defect. Two lines extending from the center of the circle to the intersections between the defect line and the circle defined the defect angle α.

##### Contralateral-Reference Method (CRM)

**Figure 1:**
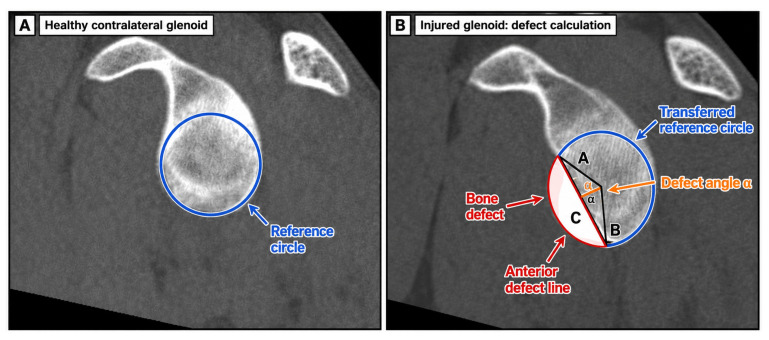
Illustration of the glenoid arc-angle measurement technique. (**A**) On the healthy contralateral glenoid, a reference circle is fitted to the intact glenoid contour. (**B**) The reference circle is transferred to the injured glenoid. The anterior osseous defect is outlined by a line connecting the superior and inferior cortical transition points. The angle α is formed by two radii extending from the center of the circle to the intersections of the defect line with the circle. A and B represent the two radii of the transferred reference circle, C represents the chord corresponding to the anterior defect line, and α denotes the central defect angle subtended by chord C. The shaded segment corresponds to the area of glenoid bone loss.

Based on the mathematical-geometrical approach described by Wambacher and Dumont [14,18], multiplanar two-dimensional reconstruction of the primary axial CT data was used to obtain an optimal en-face view of the glenoid. A best-fit circle was placed on the inferior portion of the healthy contralateral glenoid (Figure 1).

The defect could then be expressed as a percentage of the total circle area using the following formula:

$$C=\frac{\alpha -sin\alpha }{2\times \pi }\times 100$$

##### Calculation of Area-Based Glenoid Bone Loss

For every patient, observer, and measurement session, the defect angle was converted into percentage glenoid bone loss according to:

Glenoid bone loss (%) = [(α − sin α)/(2π)] × 100,

where α represents the defect angle expressed in radians. The percentage defect area was considered the final area-based measurement outcome, whereas the defect angle and glenoid diameter were retained as geometric construction parameters.

##### Injured-Side Method (ISM)

In the injured-side method, the same geometrical approach was used; however, the diameter of the best-fit circle was determined directly on the injured glenoid rather than transferred from the healthy contralateral side. For both methods, the defect angle and glenoid diameter were recorded as primary geometric measurements. Percentage glenoid bone loss was subsequently derived from each individual defect-angle measurement and was considered the final area-based outcome. The CT-based geometrical measurement workflow is illustrated in Figure 1.

### 2.5. Statistical Analysis

Statistical analyses were performed using IBM SPSS Statistics, version 29.0 (IBM Corp., Armonk, NY, USA). Intraobserver and interobserver reliability were assessed using intraclass correlation coefficients (ICCs) with 95% confidence intervals. A two-way mixed-effects, absolute-agreement, single-measure ICC model was selected. A two-way model was appropriate because all patients were evaluated by the same predefined observers and, for the intraobserver analysis, at the same two measurement sessions. The mixed-effects model was selected because the observers and measurement sessions represented fixed effects within the present study rather than observers or occasions randomly sampled from a larger population. Consequently, the reliability estimates apply primarily to the observer configuration evaluated in this study. Absolute agreement was selected because systematic differences in the magnitude of measurements between observers or sessions were considered measurement disagreement. A consistency model would retain reliability despite systematic shifts between measurements and was therefore not considered appropriate for the present objective.

Single-measure ICCs were used for the intraobserver analyses because these estimates concern one measurement session rather than an average across sessions. For the interobserver analysis, the T1 and T2 values were first averaged within each observer, and a two-way mixed-effects, absolute-agreement, single-measure ICC was then calculated across the three observer-specific means. Accordingly, the interobserver ICC estimates the reliability of a single observer-specific two-session mean rather than the reliability of a single-session measurement. Averaging the two sessions was used to reduce within-observer session variability and to focus the interobserver analysis on systematic differences among observers. The interobserver estimate is therefore labeled throughout the manuscript as the interobserver ICC of observer-specific two-session mean estimates. This model selection and reporting approach followed current methodological recommendations requiring explicit specification of the ICC model, type, and agreement definition [23,24,25]. ICCs were calculated separately for defect angle and glenoid diameter. Calculated percentage glenoid bone loss was summarized descriptively and evaluated for absolute agreement using Bland–Altman analysis, SEM, and MDC_95_. Intraobserver reliability was calculated separately for each observer by comparing the measurements obtained at the first and second measurement sessions.

ICC values are reported with 95% confidence intervals. ICC point estimates were descriptively categorized as poor when <0.50, moderate when ≥0.50 and <0.75, good when ≥0.75 and ≤0.90, and excellent when >0.90 [26,27,28]. Accordingly, an ICC of exactly 0.900 was categorized as good rather than excellent. These categories were applied only to the point estimates and were not interpreted as definitive classifications of reliability. Each ICC estimate was interpreted together with its corresponding 95% confidence interval, calculated using the same ICC model. When the confidence interval crossed one or more category boundaries, the full range of plausible reliability was reported rather than assigning an unequivocal reliability category. Absolute agreement between the first and second measurement sessions was additionally evaluated separately for each observer and measurement method using Bland–Altman analysis. Paired differences were defined as the second-session measurement minus the first-session measurement (T2 − T1); therefore, a positive value indicated a higher measurement at the second session. Mean bias and its 95% confidence interval were calculated together with the 95% limits of agreement. The limits of agreement were defined as:

$$mean\;bias\pm 1.96\times {SD}_{difference}$$

The standard error of measurement was calculated as:

$$SEM=\frac{{SD}_{difference}}{\sqrt{2}}$$

and the minimum detectable change at the 95% confidence level was calculated as:

$$MDC_{95}=1.96\times {SD}_{difference}$$

Bland–Altman analyses, SEM values, and MDC_95_ values were calculated separately for defect angle, glenoid diameter, and calculated percentage glenoid bone loss. A systematic session-related bias was considered present when the 95% confidence interval of the mean difference did not include zero.

## 3. Results

### 3.1. Demographics

A total of 31 patients (26 male and 5 female) with a mean age of 29 years (standard deviation [SD] 9.90 years) met the inclusion criteria. Of these, 21 patients underwent a J-bone graft procedure and 10 underwent screw fixation of an osseous glenoid fragment (Table 1).

### 3.2. Glenoid Measurements

The mean defect angles, glenoid diameters, and calculated area-based glenoid bone-loss percentages obtained using both geometrical approaches are presented in Table 2. The percentage defect area was calculated separately from each individual defect-angle measurement before determining the mean and standard deviation. Across observers and measurement sessions, mean glenoid bone loss ranged from 13.2% to 13.9% using the contralateral-reference method and from 15.2% to 18.6% using the injured-side method.

### 3.3. Intraobserver Reliability

For defect-angle measurements using the contralateral-reference method, the intraobserver ICC point estimates were 0.874, 0.870, and 0.826 for observers 1–3, respectively. All three point estimates were within the good reliability range. However, the corresponding 95% confidence intervals were 0.756–0.937, 0.748–0.935, and 0.672–0.912, indicating uncertainty ranging from good to excellent for observer 1 and from moderate to excellent for observers 2 and 3. Using the injured-side method, the defect-angle ICC point estimates were 0.466, 0.850, and 0.661 for observers 1–3, respectively, corresponding descriptively to poor, good, and moderate reliability. The respective 95% confidence intervals were 0.086–0.715, 0.714–0.924, and 0.385–0.825. These intervals extended from poor to moderate for observer 1, from moderate to excellent for observer 2, and from poor to good for observer 3. For glenoid-diameter measurements using the contralateral-reference method, the intraobserver ICC point estimates were 0.964, 0.953, and 0.900 for observers 1–3, respectively. The values for observers 1 and 2 were descriptively classified as excellent, whereas the value of exactly 0.900 for observer 3 was classified as good. The corresponding 95% confidence intervals were 0.910–0.984, 0.813–0.982, and 0.758–0.955, indicating excellent reliability throughout the interval for observer 1 and uncertainty ranging from good to excellent for observers 2 and 3. Using the injured-side method, the glenoid-diameter ICC point estimates were 0.735, 0.906, and 0.761 for observers 1–3, respectively. These point estimates were descriptively classified as moderate, excellent, and good. However, the corresponding 95% confidence intervals were 0.445–0.874, 0.816–0.953, and 0.232–0.910, spanning poor to good reliability for observer 1, good to excellent reliability for observer 2, and poor to excellent reliability for observer 3. Overall, the contralateral-reference method yielded numerically higher intraobserver ICC point estimates for most comparisons. Nevertheless, several confidence intervals crossed multiple reliability categories and partially overlapped between methods; therefore, the results should be interpreted descriptively rather than as evidence of statistically established superiority (Table 3).

### 3.4. Interobserver Reliability of Observer-Specific Two-Session Mean Estimates

The interobserver analyses were based on each observer’s mean of T1 and T2 and therefore quantify agreement among observer-specific two-session mean estimates rather than among single-session measurements. For defect-angle measurements, the interobserver ICC was 0.890 with the contralateral-reference method (95% CI: 0.811–0.942) and 0.603 with the injured-side method (95% CI: 0.399–0.767). The corresponding point estimates were descriptively classified as good and moderate, respectively. For glenoid-diameter measurements, the interobserver ICC was 0.900 with the contralateral-reference method (95% CI: 0.660–0.961) and 0.812 with the injured-side method (95% CI: 0.672–0.900). According to the predefined thresholds, both point estimates were classified as good. The contralateral-reference method yielded numerically higher interobserver ICC point estimates for both parameters. However, the confidence intervals crossed reliability category boundaries and partially overlapped between methods. The results should therefore be interpreted as descriptive differences in point estimates and their uncertainty rather than as evidence of statistically established superiority.

**Figure 2:**
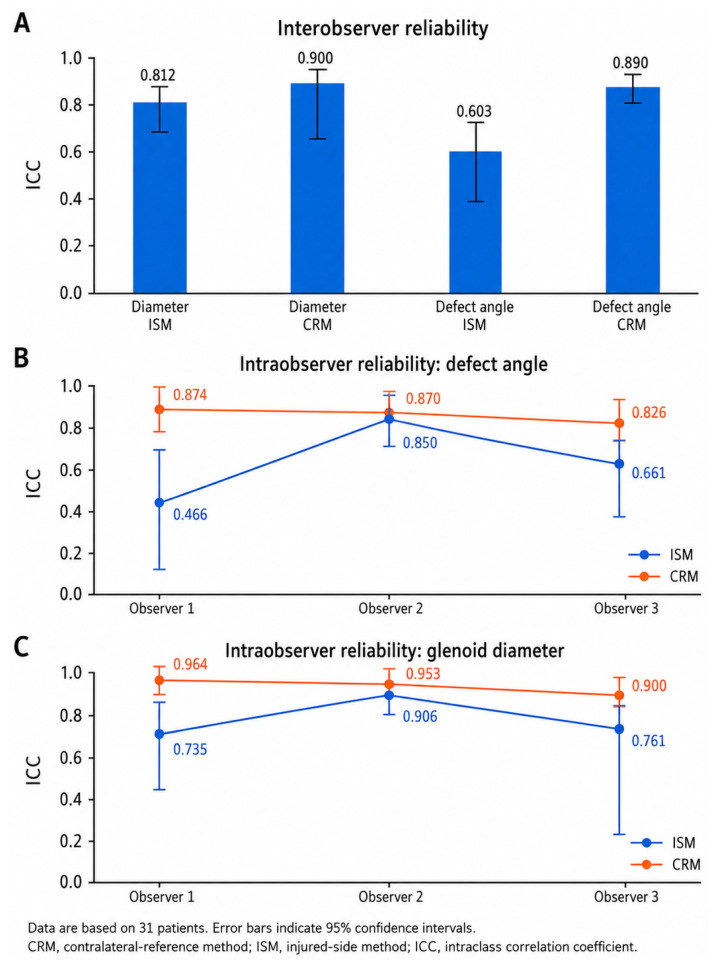
Intraobserver and interobserver reliability estimates for the injured-side and contralateral-reference methods in 31 patients. (**A**) Interobserver reliability based on observer-specific two-session mean estimates for glenoid-diameter and defect-angle measurements. (**B**) Intraobserver reliability for defect-angle measurements for observers 1–3. (**C**) Intraobserver reliability for glenoid-diameter measurements for observers 1–3. Points represent intraclass correlation coefficient point estimates, and error bars represent the corresponding 95% confidence intervals. ICC point estimates should be interpreted together with their full confidence intervals because several intervals cross conventional reliability category boundaries. CRM, contralateral-reference method; ISM, injured-side method; ICC, intraclass correlation coefficient.

To visually summarize the reliability results, ICC point estimates and their corresponding 95% confidence intervals for both measurement methods are shown in Figure 2. The contralateral-reference method showed numerically higher ICC point estimates than the injured-side method for all displayed comparisons. The largest numerical difference was observed for interobserver reliability of the defect angle, for which the ICC point estimate was 0.603 with the injured-side method and 0.890 with the contralateral-reference method. These numerical differences should be interpreted together with the corresponding confidence intervals and do not represent a formal statistical comparison between methods.

From a practical measurement perspective, the interobserver ICC point estimate for the defect angle increased by 0.287, from 0.603 with the injured-side method to 0.890 with the contralateral-reference method. For glenoid diameter, the corresponding increase was 0.088, from 0.812 to 0.900. The absolute-agreement analysis additionally showed lower measurement error for calculated percentage bone loss with contralateral referencing: the observer-specific MDC_95_ values ranged from 4.63 to 4.95 percentage points with the contralateral-reference method and from 5.65 to 8.47 percentage points with the injured-side method. These results indicate more consistent repeated measurements with contralateral referencing, particularly for the defect angle. However, the confidence intervals partially overlapped, and no predefined clinically acceptable difference was available; therefore, these findings should not be interpreted as demonstrating statistically or clinically established superiority.

### 3.5. Absolute Agreement and Measurement Error

Bland–Altman analysis yielded numerically smaller session-related differences and lower measurement-error estimates for the contralateral-reference method than for the injured-side method (Table 4). For defect-angle measurements using the contralateral-reference method, mean differences between sessions ranged from −1.27° to −0.38°, and the 95% confidence intervals included zero for all observers. The corresponding MDC_95_ values ranged from 13.48° to 15.15°. With the injured-side method, the mean differences ranged from −4.97° to 8.05°. Observer 1 demonstrated a systematic increase of 8.05° between sessions (95% CI: 3.68° to 12.42°), with limits of agreement from −15.30° to 31.40°. Observer 3 demonstrated a systematic decrease of −4.97° (95% CI: −9.01° to −0.92°). MDC_95_ values for the injured-side method ranged from 16.42° to 23.35°. For calculated percentage glenoid bone loss, mean differences using the contralateral-reference method ranged from −0.38 to 0.05 percentage points, with MDC_95_ values between 4.63 and 4.95 percentage points. Using the injured-side method, observer 1 demonstrated an increase of 2.91 percentage points (95% CI: 1.33 to 4.49), whereas observer 3 demonstrated a decrease of −1.73 percentage points (95% CI: −3.17 to −0.29). The corresponding MDC_95_ values ranged from 5.65 to 8.47 percentage points.

## 4. Discussion

The principal finding of the present study was that the contralateral-reference method yielded numerically higher intraobserver and interobserver ICC point estimates, smaller session-related biases, and lower measurement-error estimates than the injured-side method. The defect angle and glenoid diameter represent the geometric measurement components of the method, whereas the percentage defect area derived from the defect angle represents the final area-based outcome. The largest numerical difference was observed for the interobserver assessment of the defect angle based on observer-specific two-session means, for which the ICC point estimate was 0.603 with the injured-side method and 0.890 with the contralateral-reference method. Because no formal statistical comparison between methods was performed and several confidence intervals partially overlapped, these results should not be interpreted as demonstrating statistically established superiority.

The ICC point estimates should nevertheless be interpreted together with their corresponding 95% confidence intervals. In particular, the interobserver ICC for glenoid diameter obtained with the contralateral-reference method was exactly 0.900 and was therefore classified as good according to the predefined threshold, which classified only values greater than 0.90 as excellent. Moreover, its 95% confidence interval ranged from 0.660 to 0.961, spanning moderate to excellent reliability. This result therefore cannot be described unequivocally as demonstrating excellent reliability. Several other confidence intervals also crossed more than one reliability category. Accordingly, the present results should be interpreted on the basis of the ICC point estimates, their full confidence intervals, and the complementary absolute-agreement analyses rather than categorical terminology alone [26,27,28].

Importantly, greater reproducibility does not necessarily imply greater anatomical or diagnostic accuracy. Reliability describes the consistency with which a measurement can be reproduced, whereas accuracy describes the degree to which the measurement corresponds to the true anatomical defect. The higher ICC point estimates, smaller session-related biases, and lower measurement-error estimates observed with the contralateral-reference method therefore indicate greater measurement consistency within the present cohort but do not demonstrate that this approach provides a more accurate estimate of glenoid bone loss. Because no anatomical reference standard was available, the present study was designed to assess reliability and absolute agreement and cannot establish anatomical validity or diagnostic accuracy.

The Bland–Altman analyses provided additional information that was not captured by the ICCs alone. Although ICCs describe measurement reliability relative to between-subject variability, they do not independently quantify systematic bias or absolute measurement error. Using the injured-side method, observer 1 measured defect angles that were, on average, 8.05° higher during the second session, corresponding to an increase of 2.91 percentage points in calculated glenoid bone loss. Observer 3 demonstrated a mean decrease of 4.97° between sessions, corresponding to a decrease of 1.73 percentage points. These findings demonstrate that moderate or good ICC values may coexist with systematic session-related differences.

In contrast, the contralateral-reference method produced mean differences close to zero for defect-angle and percentage bone-loss measurements across all three observers. It also yielded numerically lower standard errors of measurement and minimum detectable changes than the injured-side method. These observed values are consistent with lower repeat-measurement variability in this cohort but do not establish a statistically significant difference between methods. However, the MDC_95_ values indicate the magnitude of change required to exceed the expected measurement error statistically and should not be interpreted as validated clinical decision thresholds.

The present study should be interpreted as a method-specific reliability assessment rather than as the introduction of contralateral referencing as a novel concept. Kuberakani et al. directly compared an injured-side best-fit-circle method with a contralateral comparison method and reported higher intraobserver and interobserver reliability for the latter [16]. Milano et al. evaluated two-dimensional and three-dimensional CT measurements performed with and without reference to the contralateral shoulder and reported very good agreement among the four measurement approaches [17]. More recent studies further demonstrate that the reliability and numerical magnitude of glenoid bone-loss estimates depend on both the imaging workflow and the geometric measurement method. Min et al. compared linear, area-based, and circle-line measurements and found that area-based measurement on three-dimensional CT provided the highest interobserver reliability among the evaluated approaches. Importantly, the different methods did not yield equivalent percentage values; the mean difference between linear and area-based measurements was 4.8 percentage points and was greatest near commonly discussed clinical thresholds [29]. Tennent et al. similarly evaluated six commonly used CT-based methods against models with predefined defects and concluded that the techniques differed in accuracy and should not be considered directly interchangeable [30].

Automated and artificial-intelligence-based approaches have also been developed to reduce manual variability in glenoid reconstruction and defect quantification. Zhao et al. developed a two-stage deep-learning model for CT-based glenoid segmentation and quantitative bone-loss assessment. Their study included 237 patients with recurrent unilateral shoulder dislocation and 248 controls and demonstrated high concordance between predicted and reference measurements [31]. Haimi et al. described an automated CT pipeline incorporating selection of the glenoid en-face plane, fitting of a circle to the posteroinferior glenoid contour, and subsequent bone-loss quantification [32]. Satir et al. further reported a deep-learning approach for automated quantification of scapular and glenoid morphology from CT examinations [33]. These developments demonstrate the potential of automated workflows to standardize plane selection, segmentation, and geometric measurement. However, they use different computational and geometric definitions and do not directly validate the glenoid arc-angle method evaluated in the present study [31,32,33].

In this context, the present investigation provides complementary information about a manually applied, area-based arc-angle method that can be performed using conventional multiplanar CT reconstruction. Its principal contribution is the comparison of two reference-circle strategies and the evaluation of their reproducibility and absolute agreement. The results should not be interpreted as demonstrating that this method is more accurate than three-dimensional, segmented, or automated approaches because no direct comparison with these techniques was performed. The present investigation differs from these previous studies in its geometric outcome and measurement workflow. The glenoid arc-angle method uses the central angle formed by the anterior defect chord and the center of the reference circle to calculate the area of the corresponding circular segment [18,19]. The current analysis also included three observers with different levels of experience, repeated measurements during two sessions, and complementary assessment of systematic bias, limits of agreement, standard error of measurement, and minimum detectable change. Nevertheless, the present findings do not establish anatomical accuracy, superiority regarding treatment selection, or an association with clinical outcomes.

The potential clinical relevance of the numerically lower measurement-error estimates observed with contralateral referencing may lie in reducing observer- and session-related measurement variability, particularly when estimated bone-loss values are close to thresholds considered during clinical assessment. In such cases, lower measurement variability could theoretically reduce changes in defect classification caused solely by differences in reconstruction-plane selection, reference-circle placement, or defect-line definition. However, this interpretation remains inferential because no formal between-method comparison was performed. The present study did not evaluate predefined defect categories or determine whether the two reference-circle strategies resulted in different classifications of individual patients. It also did not assess whether contralateral referencing altered surgical indications or influenced the choice among isolated soft-tissue stabilization, remplissage, Latarjet reconstruction, or other bone-augmentation procedures. The observed numerical differences in reliability and measurement-error estimates therefore do not establish differences in glenoid-defect classification, therapeutic decision-making, or patient outcomes.

The percentage reported in the present study represents an area-based measurement, defined as the area of the circular segment corresponding to the glenoid defect relative to the total area of the reference circle [18]. This outcome differs conceptually from one-dimensional methods that express bone loss as a percentage of glenoid width. Area-based and width-based measurements describe different geometric properties and should not be considered directly interchangeable. Consequently, thresholds derived from width-based methods cannot automatically be applied to arc-angle-derived area percentages without method-specific clinical validation.

The clinical interpretation of glenoid bone loss has evolved beyond the traditional critical threshold of approximately 20–25% [4,5,7]. Shaha et al. reported poorer functional outcomes when glenoid bone loss exceeded approximately 13.5%, contributing to the concept of subcritical bone loss [6]. This observation indicates that defects below the traditional critical range may already be clinically relevant. However, the 13.5% value was derived from a specific patient population and outcome measure and should not be regarded as a universally validated surgical threshold. Furthermore, because the percentage produced by the present method is area-based, it should not be assumed to be directly equivalent to thresholds derived using width-based measurements.

Glenoid bone loss also represents only one component of bipolar bone loss. Di Giacomo, Itoi, and Burkhart integrated glenoid bone loss and Hill–Sachs morphology through the glenoid track concept [34]. An anterior glenoid defect reduces the available glenoid track, and a Hill–Sachs lesion is classified as off-track when the Hill–Sachs interval exceeds the available track. This relationship may influence the choice among isolated soft-tissue stabilization, remplissage, and glenoid bone-augmentation procedures. Reliable glenoid measurements may therefore contribute to bipolar bone-loss assessment but cannot replace evaluation of the humeral-sided lesion. The present study did not assess Hill–Sachs morphology, the Hill–Sachs interval, or on-track/off-track status and consequently evaluates only the glenoid component of this clinical framework.

Beyond conventional geometric measurements, individualized computational approaches may provide additional opportunities for quantitative orthopedic assessment. Recent studies have combined patient-specific loading information, parameterized geometries, finite element analysis, machine-learning surrogate models, and data-driven optimization to adapt structural properties to individual biomechanical requirements [35,36]. Lu et al. developed a parameterized lattice insole using finite element analysis and machine learning to investigate cushioning performance [35]. A subsequent dual-layer framework combined finite element analysis, Gaussian process regression, and Bayesian optimization to adapt lattice pads to gait-induced pressure distributions [36].

Although these studies addressed plantar biomechanics rather than shoulder instability, the underlying computational principles may be transferable to future glenoid research. Patient-specific glenoid morphology, defect size, Hill–Sachs geometry, joint loading, and reconstruction parameters could potentially be incorporated into parametric biomechanical models to simulate contact mechanics and compare alternative treatment strategies. Data-driven surrogate models could subsequently reduce the computational burden associated with testing multiple defect and reconstruction configurations. However, the present study did not include finite element modeling, machine learning, biomechanical simulation, or computational optimization. These applications therefore represent future research directions rather than conclusions directly supported by the present data.

A strength of the present study was the inclusion of observers with different levels of experience, including an experienced shoulder surgeon and two medical students. The numerical pattern of higher ICC point estimates and lower measurement-error estimates with contralateral referencing suggests that this approach may be less sensitive to observer-related variation within the controlled setting of the study, although no formal comparison according to observer experience was performed. However, the results do not demonstrate that observer experience is irrelevant, and appropriate training and standardized measurement rules remain necessary.

Another practical advantage is that the measurements can be performed using standard CT data and conventional multiplanar reconstruction and angle-measurement tools. No dedicated three-dimensional segmentation or specialized analysis software is required. This may facilitate implementation in routine clinical imaging workflows when bilateral CT data are already available and the contralateral shoulder is unaffected. Nevertheless, different CT measurement methods are not necessarily interchangeable, and reported accuracy and reliability may depend on the reconstruction procedure, geometric definition, and available anatomical reference [2,16,17].

The present study has several limitations. First, the retrospective design introduces a potential risk of selection bias because inclusion depended on the availability and completeness of previously acquired clinical records and bilateral preoperative CT examinations. The cohort consisted exclusively of surgically treated patients who underwent a bony reconstruction procedure and may therefore have been enriched for patients with more severe instability patterns or clinically relevant glenoid bone loss. Patients with smaller defects, primary instability, non-operative treatment, isolated soft-tissue stabilization, or no bilateral CT examination were not represented. The requirement for an unaffected contralateral shoulder further restricted the eligible population. Consequently, the findings should not be generalized directly to the broader population of patients with anterior shoulder instability.

Second, no formal a priori sample-size calculation based on predefined acceptable and expected ICC values was performed. The cohort size was determined by the number of eligible bilateral CT examinations available during the retrospective study period. The study should therefore be regarded as exploratory rather than as a prospectively powered confirmatory reliability investigation. The limited sample size contributed to the width of several 95% confidence intervals, some of which crossed multiple conventional reliability categories. Accordingly, the ICC point estimates are associated with substantial statistical uncertainty and should be interpreted cautiously rather than as precise evidence of a definitive difference between the two reference-circle strategies. Confirmation of these findings in a larger, prospectively planned cohort is required.

Third, the CT examinations were acquired between 2007 and 2012 using an older-generation scanner. Differences in spatial resolution, acquisition parameters, and reconstruction algorithms may limit direct transferability to contemporary CT systems. Moreover, the contralateral-reference method requires suitable imaging of both shoulders and assumes that the opposite glenoid is unaffected. Bilateral shoulder CT is not routinely performed in all institutions, and its availability may depend on local imaging protocols, clinical indications, scanner configuration, and healthcare resources. The present study did not quantify protocol-specific radiation exposure attributable to bilateral imaging and did not evaluate acquisition time, costs, cost-effectiveness, or feasibility in different clinical settings. The practical applicability of the method may therefore be greatest when bilateral CT data are already available or when imaging of the contralateral shoulder is clinically justified.

Fourth, the study evaluated measurement reliability and absolute agreement rather than anatomical validity or diagnostic accuracy. No anatomical ground truth was available, such as direct cadaveric measurement, intraoperative measurement, validated three-dimensional surface segmentation, or a reference phantom. The study therefore cannot determine whether the calculated area-based percentages overestimate or underestimate the true glenoid defect.

Fifth, the glenoid arc-angle method was not directly compared with established techniques such as the Pico method, conventional best-fit-circle measurements, the Glenoid Index, or dedicated three-dimensional and automated CT methods. The present findings therefore demonstrate differences in reproducibility between two reference-circle strategies within the arc-angle method but do not establish greater accuracy or interchangeability relative to other measurement techniques. Higher ICC point estimates and lower measurement-error estimates with the contralateral-reference method indicate greater measurement consistency but do not establish anatomical accuracy or clinical superiority. The study did not evaluate whether contralateral referencing altered surgical indications or influenced the selection of arthroscopic Bankart repair, remplissage, Latarjet reconstruction, or another bone-augmentation procedure. No associations were examined with recurrent instability, treatment failure, functional scores, patient-reported outcomes, or postoperative results. Consequently, the numerical differences in reproducibility and measurement-error estimates should not be interpreted as evidence of better therapeutic decision-making or clinical outcomes.

Finally, no predefined clinically acceptable measurement-error threshold was available. The MDC_95_ values indicate the magnitude of change required to exceed expected measurement error but should not be interpreted as validated treatment thresholds. Hill–Sachs morphology, the Hill–Sachs interval, and the glenoid track relationship were not assessed. The present results therefore cannot classify bipolar lesions as on-track or off-track or directly determine surgical treatment.

## 5. Conclusions

The CT-based glenoid arc-angle method demonstrated reproducible area-based measurement of glenoid bone loss in this selected cohort. Using the unaffected contralateral glenoid to define the reference circle yielded numerically higher ICC point estimates, smaller session-related biases, and lower absolute measurement-error estimates than constructing the reference circle directly on the injured glenoid. The largest numerical difference was observed for defect-angle measurements. Because no formal statistical comparison between methods was performed and several confidence intervals partially overlapped, these findings do not establish statistically significant superiority. They are limited to measurement reliability and absolute agreement and do not establish anatomical accuracy, diagnostic validity, clinical superiority, or an effect on therapeutic decision-making or patient outcomes. The area-based percentages generated by the glenoid arc-angle method should not be considered directly interchangeable with values or treatment thresholds derived using other geometric methods. The observed numerical differences require confirmation in larger, prospectively planned studies using contemporary imaging, anatomical or three-dimensional reference standards, and clinical outcome data.

## Figures and Tables

**Table 1:** Demographic and clinical characteristics of the study cohort (*n* = 31).

Variable	Value
Age, years, mean ± SD	29.0 ± 9.9
Male sex, *n* (%)	26 (83.9)
Female sex, *n* (%)	5 (16.1)
Right shoulder affected, *n* (%)	18 (58.1)
Left shoulder affected, *n* (%)	13 (41.9)
J-bone graft procedure, *n* (%)	21 (67.7)
Screw fixation of an osseous glenoid fragment, *n* (%)	10 (32.3)

Values are presented as *n* (%) unless otherwise indicated. SD, standard deviation.

**Table 2:** Defect angle, glenoid diameter, and calculated area-based glenoid bone loss according to measurement method, observer, and measurement session in the study cohort (*n* = 31).

Parameter	Method	Observer	T1, Mean ± SD	T2, Mean ± SD
Defect angle, °	CRM	1	104.3 ± 13.2	103.7 ± 13.9
	CRM	2	102.2 ± 13.0	101.9 ± 15.7
	CRM	3	104.2 ± 12.6	102.9 ± 13.5
	ISM	1	109.2 ± 14.3	117.2 ± 10.6
	ISM	2	109.1 ± 16.0	107.5 ± 14.6
	ISM	3	114.9 ± 12.7	110.0 ± 15.4
Glenoid diameter, mm	CRM	1	27.6 ± 2.2	27.3 ± 2.2
	CRM	2	28.0 ± 2.0	28.4 ± 2.1
	CRM	3	28.3 ± 2.3	27.9 ± 2.0
	ISM	1	27.8 ± 2.5	28.9 ± 2.9
	ISM	2	28.9 ± 2.7	28.7 ± 2.4
	ISM	3	29.8 ± 2.7	28.5 ± 2.6
Glenoid bone loss, %	CRM	1	13.9 ± 4.6	13.8 ± 4.9
	CRM	2	13.2 ± 4.4	13.3 ± 5.3
	CRM	3	13.9 ± 4.5	13.5 ± 4.6
	ISM	1	15.7 ± 5.5	18.6 ± 4.4
	ISM	2	15.8 ± 6.0	15.2 ± 5.5
	ISM	3	17.8 ± 5.1	16.1 ± 5.7

Values are presented as mean ± standard deviation. Percentage glenoid bone loss was calculated separately from each individual defect-angle measurement. CRM, contralateral-reference method; ISM, injured-side method; T1, first measurement session; T2, second measurement session; SD, standard deviation.

**Table 3:** Intraobserver and interobserver reliability of glenoid defect measurements. Intraclass correlation coefficients are shown for defect-angle and glenoid-diameter measurements using the injured-side and contralateral-reference methods. Intraobserver reliability was calculated separately for each observer by comparing T1 and T2. Interobserver reliability was calculated across the three observer-specific means of T1 and T2 and therefore represents agreement among two-session mean estimates rather than among single-session measurements. Higher ICC point estimates indicate greater estimated measurement reliability but do not establish a statistically significant difference between methods.

Parameter	Reliability	Observer	ISM ICC (95% CI)	Classification	CRM ICC (95% CI)	Classification
Glenoid diameter	Intraobserver	1	0.735 (0.445–0.874)	Moderate	**0.964 (0.910–0.984)**	Excellent
		2	0.906 (0.816–0.953)	Excellent	**0.953 (0.813–0.982)**	Excellent
		3	0.761 (0.232–0.910)	Good	**0.900 (0.758–0.955)**	Good
Defect angle	Intraobserver	1	0.466 (0.086–0.715)	Poor	**0.874 (0.756–0.937)**	Good
		2	0.850 (0.714–0.924)	Good	**0.870 (0.748–0.935)**	Good
		3	0.661 (0.385–0.825)	Moderate	**0.826 (0.672–0.912)**	Good
Glenoid diameter	Interobserver	All	0.812 (0.672–0.900)	Good	**0.900 (0.660–0.961)**	Good
Defect angle	Interobserver	All	0.603 (0.399–0.767)	Moderate	**0.890 (0.811–0.942)**	Good

ICC point estimates were descriptively classified as poor when <0.50, moderate when ≥0.50 and <0.75, good when ≥0.75 and ≤0.90, and excellent when >0.90. Bold formatting indicates the numerically higher ICC point estimate and its corresponding 95% confidence interval within each paired ISM–CRM comparison. It does not indicate a statistically significant difference between methods. Classifications are based on point estimates only; all ICCs should be interpreted together with their full 95% confidence intervals. No *p*-values are reported because no formal statistical comparison of ICCs was performed. CRM, contralateral-reference method; ISM, injured-side method; ICC, intraclass correlation coefficient; CI, confidence interval.

**Table 4:** Bland–Altman analysis and absolute measurement error between the two measurement sessions.

Parameter	Method	Observer	Mean Bias, T2 − T1 (95% CI)	95% Limits of Agreement	SEM	MDC_95_
Defect angle, °	CRM	1	−0.66 (−3.18 to 1.87)	−14.14 to 12.83	4.86	13.48
Defect angle, °	CRM	2	−0.38 (−3.11 to 2.35)	−14.97 to 14.22	5.26	14.59
Defect angle, °	CRM	3	−1.27 (−4.11 to 1.56)	−16.43 to 13.88	5.47	15.15
Defect angle, °	ISM	1	8.05 (3.68 to 12.42)	−15.30 to 31.40	8.42	23.35
Defect angle, °	ISM	2	−1.55 (−4.62 to 1.53)	−17.97 to 14.88	5.93	16.42
Defect angle, °	ISM	3	−4.97 (−9.01 to −0.92)	−26.58 to 16.64	7.80	21.61
Glenoid diameter, mm	CRM	1	−0.27 (−0.46 to −0.07)	−1.30 to 0.77	0.37	1.03
Glenoid diameter, mm	CRM	2	0.39 (0.20 to 0.58)	−0.63 to 1.40	0.37	1.01
Glenoid diameter, mm	CRM	3	−0.46 (−0.78 to −0.15)	−2.17 to 1.24	0.62	1.71
Glenoid diameter, mm	ISM	1	1.02 (0.36 to 1.68)	−2.52 to 4.56	1.28	3.54
Glenoid diameter, mm	ISM	2	−0.21 (−0.62 to 0.21)	−2.41 to 2.00	0.80	2.20
Glenoid diameter, mm	ISM	3	−1.32 (−1.84 to −0.79)	−4.13 to 1.50	1.02	2.82
Glenoid bone loss, %	CRM	1	−0.19 (−1.05 to 0.68)	−4.82 to 4.44	1.67	4.63
Glenoid bone loss, %	CRM	2	0.05 (−0.82 to 0.92)	−4.62 to 4.71	1.68	4.66
Glenoid bone loss, %	CRM	3	−0.38 (−1.31 to 0.55)	−5.33 to 4.57	1.78	4.95
Glenoid bone loss, %	ISM	1	2.91 (1.33 to 4.49)	−5.56 to 11.37	3.05	8.47
Glenoid bone loss, %	ISM	2	−0.66 (−1.72 to 0.40)	−6.31 to 4.99	2.04	5.65
Glenoid bone loss, %	ISM	3	−1.73 (−3.17 to −0.29)	−9.43 to 5.97	2.78	7.70

Positive bias indicates a higher value at the second measurement session. CRM, contralateral-reference method; ISM, injured-side method; SEM, standard error of measurement; MDC_95_, minimum detectable change at the 95% confidence level.

## Data Availability

The data presented in this study are available on request from the corresponding author. The data are not publicly available due to ethical restrictions.

## Abbreviations

The following abbreviations are used in this manuscript:

CT	Computed tomography
CRM	Contralateral-reference method
ISM	Injured-side method
ICC	Intraclass correlation coefficient
SD	Standard deviation
CI	Confidence interval
SPSS	Statistical Package for the Social Sciences
